# Graded activation of mutant K41C-KCNE1:KCNQ1 channel complexes by mefenamic acid

**DOI:** 10.1080/19336950.2025.2539494

**Published:** 2025-07-29

**Authors:** Yundi Wang, Magnus Chan, Marc Pourrier, Jodene Eldstrom, David Fedida

**Affiliations:** Department of Anesthesiology, Pharmacology and Therapeutics, University of British Columbia, Vancouver, BC, Canada

**Keywords:** Ion channels, mefenamic acid, *I*_Ks_

## Abstract

The *I*_*Ks*_ current formed by the co-assembly of KCNE1 and KCNQ1 plays an important role in cardiac repolarization. Mefenamic acid, an NSAID, is known to enhance *I*_*Ks*_ currents and has in turn been suggested as a therapeutic starting point for the development of compounds for the treatment of long QT syndrome. Our previous examinations of mefenamic acid’s action revealed that residue K41 on KCNE1 was critical for mefenamic acid’s activating effect on fully KCNE1 saturated, and partially saturated *I*_*Ks*_ channel complexes. The present study extends our previous work by incorporating the K41C-KCNE1 mutation into individual subunits to destabilize local mefenamic acid binding and explore how many of the remaining mefenamic acid-bound WT KCNE1-KCNQ1 subunits are required to support the activating action of the drug. Our results show that the potency of mefenamic acid action is reduced by the presence of K41C-KCNE1 subunits in a graded and stoichiometric, but non-linear manner. Modeling results are consistent with the idea that WT *I*_*Ks*_ subunits, in the presence of mefenamic acid, precede activation of K41C-*I*_*Ks*_ subunits due to their augmented voltage sensor kinetics.

## Introduction

Varying numbers of one to four KCNE1 subunits have been shown to co-assemble with KCNQ1, and while the exact stoichiometric ratio of subunits comprising the delayed cardiac rectifier potassium current, I*_Ks_*, remains unknown in humans, activators which are effective on all *I*_*Ks*_ stoichiometric ratios are of interest [1–5]. Most known activators of *I*_*Ks*_ such as ML277, zinc pyrithione, and L-364,373, unfortunately, are only effective on KCNQ1 alone with limited efficacy on increasingly saturated *I*_*Ks*_ channel complexes [6–8].

Of interest, mefenamic acid, a nonsteroidal anti-inflammatory drug (NSAID), has previously been identified by numerous groups to enhance *I_Ks_* currents in various expression systems including canine and guinea-pig ventricular myocytes [8,9] as well as heterologous expression systems such as *Xenopus laevis* oocytes and CHO, COS-7, tsA201, and LM cells [9–13]. After treating *I*_*Ks*_ with 100 µM mefenamic acid, variable amounts of instantaneous current and inhibition of tail current decay were reported. In our characterization of the effects of mefenamic acid on *I*_*Ks*_, residue K41, located on the extracellular end of KCNE1, was found to be critical in mediating the activating effect of mefenamic acid on the fully saturated *I*_*Ks*_ channel complex (4:4 ratio of KCNE1 to KCNQ1, EQ) [13]. Cysteine scanning revealed that although other extracellular KCNE1 residues in the same region to varying degrees impacted the effect of mefenamic acid, only the K41C mutation completely abolished mefenamic acid effects up to a concentration of 1 mM. Previous cross-linking studies have identified key interactions between this extracellular region of KCNE1 and the transmembrane segments, S6 and S1 of KCNQ1, suggesting that residues in either the S6 and/or S1 regions could also provide critical clues to explain the mechanism of action of mefenamic acid [14]. Building on this, our recent study has identified a generalized activator binding pocket for mefenamic acid that is formed extracellularly by KCNE1, the domain-swapped S1 helices of one KCNQ1 subunit and the pore/turret region made up of two other KCNQ1 subunits [15]. Particularly K41 in KCNE1, but also other residues such as W323, in the adjacent KCNQ1 subunits appear critical for mefenamic acid binding.

A remaining unresolved question is how many mefenamic acid binding sites need to be occupied to permit the activating action of the drug on KCNQ1-KCNE1 complexes, and how this affects voltage sensor (VS) transitions in the activation pathway. To address this, we have made fixed-stoichiometry *I*_*Ks*_ mutants containing K41C residues in different numbers of the four KCNE1 accessory subunits. We find that reduction in the number of available mefenamic acid binding sites reduces the activating response to the drug, and the presence of K41C in more than one KCNE1 subunit greatly diminishes the response to 100 μM mefenamic acid.

## Materials and methods

### Reagents and solutions

Mefenamic acid (Tocris Bioscience, Oakville, ON, Canada) was used at concentrations of 10 μM, 30 μM, 100 μM, 300 μM, 500 μM, and 1 mM. To block *I*_*Ks*_, the specific *I*_*Ks*_ inhibitor HMR1556, (3 R,4S)-(+)-N-[3-hydroxy-2,2-dimethyl-6-(4,4,4-trifluorobutoxy) chroman-4-yl]-N-methylmethanesulfonamide (Tocris Bioscience, Oakville, ON, Canada) was used at a concentration of 1 µM. All other reagents and solutions were prepared as previously described [13].

### Molecular biology, cell culture, and whole cell patch clamp

All mutations were generated using site-directed mutagenesis and confirmed by sequencing. tsA201 transformed human embryonic kidney 293 cells were first cultured, then plated on coverslips for whole-cell experiments and, finally, transfected using Lipofectamine2000 as previously described [3,13,16]. Whole-cell experiments were conducted 24–48 hour post transfection. For wildtype (WT) EQ and mutant x-EQ, where “x” denotes a KCNE1 mutation for example, K41C-EQ, cells were transfected with a linked KCNE1 and KCNQ1 cDNA (1.25 µg was used) which assembles as a fully saturated 4:4 ratio of KCNE1 to KCNQ1. For experiments where mutant K41C-KCNE1s were introduced into one (1:3 ratio of mutant to WT KCNE1), two (2:2 ratio of mutant to WT KCNE1), and three (3:1 ratio of mutant to WT KCNE1) out of the four β-subunits in a fully saturated *I*_*Ks*_ complex, cells were transfected with the following constructs: for the 1:3 ratio of mutant to WT KCNE1 *I*_*Ks*_ construct (K41C-EQQQQ + WT KCNE1), cells were co-transfected in an 9:2.25 µg ratio of WT KCNE1-GFP to K41C-EQQQQ (K41C-KCNE1 linked with four KCNQ1 subunits, which assembles as a 1:4 ratio of KCNE1 to KCNQ1); for the 2:2 ratio of mutant to WT KCNE1 *I*_*Ks*_ construct (EQQ + K41C-KCNE1), cells were co-transfected in an 8:2 µg ratio of mutant K41C-KCNE1 to EQQ (where WT KCNE1 is linked to a KCNQ1 dimer, which assembles as a 2:4 ratio of KCNE1 to KCNQ1); for the 3:1 ratio of mutant to WT KCNE1 *I*_*Ks*_ construct (EQQQQ + K41C-KCNE1), cells were co-transfected in an 9:2.25 µg ratio of mutant K41C-KCNE1 to EQQQQ (WT KCNE1 linked to four KCNQ1 subunits, which assembles as a 1:4 ratio of KCNE1 to KCNQ1). While we cannot completely exclude the possibility that a small fraction of unsaturated channels may exist, our coexpression strategy follows previous work from our group. When excess KCNE1 is coexpressed with Q1 (including EQQ, EQQQQ, and WT Q1 constructs), the resulting channel complexes exhibit indistinguishable voltage-dependence of activation [3]. This convergence implies that available KCNE1 subunits can be effectively incorporated into the channel complex, yielding a saturated 4:4 stoichiometry under our experimental conditions. Constructs other than WT KCNE1 were also co-transfected with 0.8 µg of GFP to allow transfected cells to be identified. Data were obtained using an Axopatch 200B amplifier, a Digidata 1440A digitizer, and pCLAMP 11 software.

### Data analysis

The normalized response to increasing concentrations of mefenamic acid was calculated in the same manner as previously described [13]. While this measurement captures the increase in instantaneous current induced by mefenamic acid, it is important to note that any inhibition occurring at high drug concentrations is not reflected in the normalized response, as this calculation accounts only for changes in current waveform morphology, not current amplitude. In these experiments, each cell was exposed to one or more drug concentrations, as long as cell health and recording quality remained stable. Current potentiation at each concentration resulted in concentration–response relationships which were fitted to the following equation: $\frac{I}{I_{\max}}=\frac{1}{1+{\left(\frac{EC_{50}}{\left[A\right]}\right)}^{n_{H}}}$ to obtain the EC_50_, which is the concentration of mefenamic acid that gives a response halfway between the lower and upper asymptotes of the dose–response curves (i.e. the half maximal effective concentration). The Hill coefficient (n_H_) was fixed to 1, consistent with the assumption of noncooperative binding behavior expected from a simple mass action interaction model between mefenamic acid and its binding site (1:1 stoichiometry). While the bottom of the curves was constrained to 0 (no drug effects), no constraint was applied to the top of the curves.

Where applicable, one-way ANOVA followed by the Bonferroni multiple comparison post-hoc test was used to determine statistical significance. In all figures ****, ***, **, * denote significance where *p* < 0.0001, *p* < 0.0005, *p* < 0.01, and *p* < 0.05, respectively. All data in the figures and tables are shown as mean ± SEM. Statistical analysis was done in Graphpad Prism 9. For EC_50_s, the 95% confidence interval is reported.

### Modeling

IonChannelLab software [17] was used to model *I*_*Ks*_ in Figure 3, as we did previously [16] and in modeling the action of mefenamic acid on wild type (WT) *I*_Ks_ channels [13]. In this model, KCNQ1:KCNE1 in a 4:4 stoichiometry assumes four VS each undergoes two activating transitions, to an intermediate and then activated conformation. Pore subconductance opening can occur as soon as each VS is fully activated, and thus pore opening does not require a concerted step after all four VSs have reached fully activated conformations. For full model exposition and rates, see the supplemental materials for Westhoff et al. 2019 [16]. As in our prior study, to simulate the action of mefenamic acid on *I*_*Ks*_ currents, the intrinsic rates of forward VS transitions at 0 mV, between resting and intermediate states (k_RI_0) and between intermediate and activated states (k_IA_) were multiplied by the drug concentration (D, in µM), or log_10_ [D], respectively [13].

## Results

### Mefenamic acid actions are prevented by the KCNE1 mutation, K41C in a stoichiometric manner

**Figure 1.**
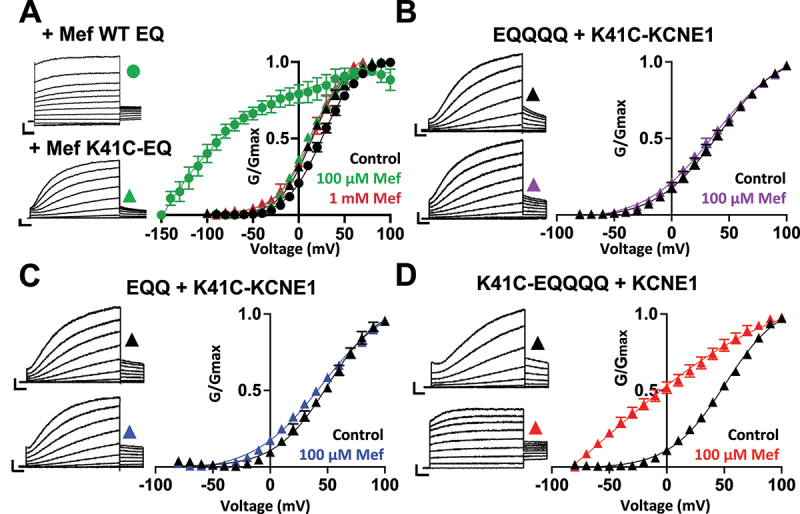
K41C-KCNE1 mutants prevent the agonist effect of mefenamic acid.

**Figure 2.**
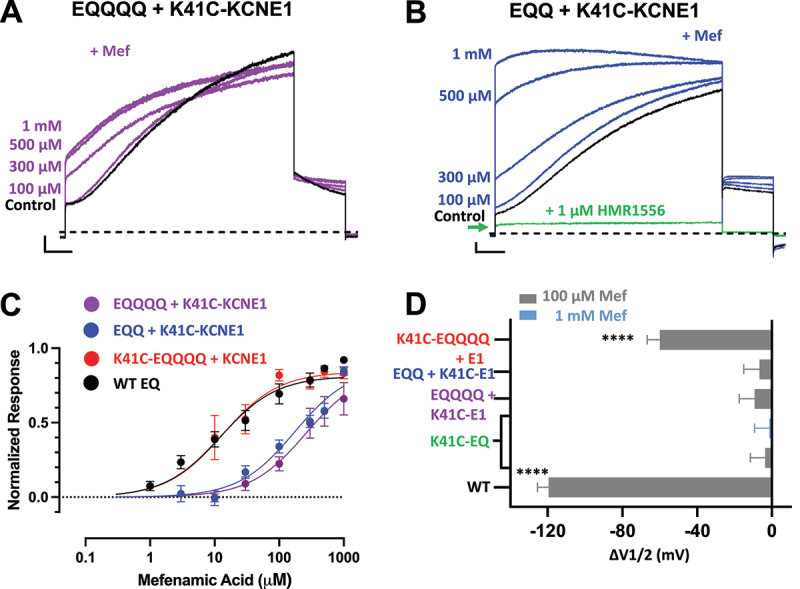
Stoichiometric prevention of mefenamic acid agonism by K41C-KCNE1 mutants.

Consistent with our previous study and those of others [11–13], treatment of fully saturated WT *I*_*Ks*_ complexes (4:4 ratio; WT EQ) with 100 µM mefenamic acid transformed the slowly activating *I*_*Ks*_ current and deactivating tail current into one with an almost linear waveform indicating instantaneous current onset and complete inhibition of tail current decay (Figure 1A, top left). The corresponding conductance-voltage (G-V) relationship obtained from initial tail currents shows, in the presence of 100 µM mefenamic acid, a hyperpolarization (difference in voltage at half-maximal activation, ΔV_1/2_ = −119.4 mV) and shape change (decreased slope) of the G-V relationship compared to control (control *k*, slope factor = 19.4 mV; Mef *k*, slope factor = 36.7 mV) (Figure 1A, right, black vs green circles, and Table 1). As the drug concentration increases, the instantaneous current observed at the start of the step pulse becomes progressively larger, resulting in a flatter overall current waveform. The corresponding dose–response curve based on normalized response (see Materials and Methods for calculation) approaches a value of 1.0 (Figure 2C). This approach captures the functional impact of mefenamic acid on macroscopic currents while limiting recording times and avoiding issues related to current rundown and the potential introduction of large variations in V_1/2_ measurements. In our previous study, we also reported that at higher drug concentrations, a reduction in the current amplitude becomes evident during the step pulse and tail current, indicating a second effect of channel block at very high concentrations [18]. Thus, while the normalized response metric is useful for comparing potentiation effects across constructs and concentrations, it does not capture changes in absolute current amplitude and therefore does not measure the extent of inhibition.

(A) WT EQ currents (top) and K41C-EQ (bottom) in 100 µM mefenamic acid (Mef). G-V plot from WT EQ tail currents (circles) and K41C-EQ (triangles) in the absence (control: black) and presence of Mef (100 µM: green; 1 mM: red) (right). Boltzmann fit parameters are in Table 1. Scale bars in all panels represent 0.5 nA and 0.5 s. (B-D) Current traces of EQQQQ + K41C-KCNE1 (B), EQQ + K41C-KCNE1 (C), and K41C-EQQQQ + KCNE1 (D) in control (top) and 100 µM Mef (bottom). G-V plots are in control (black) and 100 μM Mef (colored). The Boltzmann fit parameters are in Table 1. WT EQ, K41C-EQ, EQQQQ + K41C-KCNE1, EQQ + K41C-KCNE1 and K41C-EQQQQ + KCNE1 *I*_*Ks*_ constructs have a WT KCNE1:K41C-KCNE1 ratio of 4:0, 0:4, 1:3, 2:2 and 3:1, respectively. A 4 s protocol with pulses from -150 mV or higher to +100 mV followed by a repolarization step of -40 mV for 1 s was used. Holding potential and inter-pulse interval were -80 mV and 15 s, respectively.

(A and B) Currents (control: black) exposed to increasing concentrations of Mef (EQQQQ + K41C-KCNE1: purple; EQQ + K41C-KCNE1: blue), and in B, 1 µM HMR1556 (green). Complete inhibition by HMR1556 is indicated by the green arrow. Scale bars represent 0.5 nA and 0.5 s. (C) Mean log concentration-response curves with Hill coefficient (n_H_) constrained to 1.0 for EQQQQ + K41C-KCNE1 (purple circles, *n* = 4–10 at each concentration; EC_50_ = 262 [124, 593; 95% CI] μM; EQQ + K41C-KCNE1 (blue circles, *n* = 3–12 at each concentration; EC_50_ = 173 [89.0, 356; 95% CI] μM) and; K41C-EQQQQ + KCNE1 (red circles, *n* = 2–5 at each concentration; EC_50_ = 13.3 [6.14, 25.9; 95% CI] μM); and, WT EQ (black circles, *n* = 2–7 at each concentration: EC_50_ = 12.4 [6.82, 21.5; 95% CI] μM). A 4 s protocol to +60 mV followed by a repolarization step to -40 mV for 1 s was used to obtain currents (see Methods for calculation of normalized response). Holding potential and interpulse interval were -80 mV and 15 s, respectively. (D) Summary plot of V_1/2_ change (Δ V_1/2_) in the presence of 100 µM mefenamic acid. **** denotes a significant Δ V_1/2_ compared to control where *p* < 0.0001. ΔV_1/2_ were obtained from data in Figure 1.

**Table 1.** V_1/2_ of activation and slope factor (*k*) for Boltzmann fits in the absence and presence of mefenamic acid for fully saturated *I*_*Ks*_ channel complexes. p-value indicates statistical difference between V_1/2_ in drug compared to control, determined using a one-way ANOVA and Bonferroni multiple comparisons test. NS denotes “not significant.” Values are shown as mean ± SEM.

	Control			100 µM or 1 mM Mefenamic Acid *			p-value
	V_1/2_ (mV)	*k (mV)*	n	V_1/2_ (mV)	*k (mV)*	n	
Wildtype (WT) EQ	25.7 ± 2.6	19.4 ± 1.2	6	−93.8 ± 12	36.7 ± 14.2	5	<0.0001
EQQQQ + K41C-KCNE1	44.0 ± 2.2	27.2 ± 1.3	4	34.8 ± 2.6	30.4 ± 2.8	3	NS
EQQ + K41C-KCNE1	52.0 ± 2.3	23.7 ± 1.6	4	45.3 ± 2.1	29.8 ± 1.4	3	NS
K41C-EQQQQ + KCNE1	57.6 ± 1.5	27.6 ± 1.9	4	−2.38 ± 4.7	44.5 ± 5.8	4	<0.0001
K41C-EQ	17.0 ± 1.6	19.6 ± 1.6	7	11.7 ± 1.0	18.6 ± 0.6	3	NS
				16.7 ± 2.0	19.8 ± 1.4	3	NS

*For K41C-EQ, the concentration of mefenamic acid used was either 100 µM (upper row values) or 1 mM (lower row values). For all other constructs 100 µM mefenamic acid was used.

The introduction of a cysteine mutation at residue K41 in all four KCNE1 subunits (4:4 ratio of mutant K41C-KCNE1 to KCNQ1; K41C-EQ) abolished all changes to the G-V relationship and current waveform up to a concentration of 1 mM mefenamic acid. No significant shift in the V_1/2_ or changes to the slope of the G-V plot were seen after treatment with not only 100 µM but also 1 mM mefenamic acid (Figure 1A, right, triangles, and Table 1). Additionally, the current waveforms of K41C-EQ remained sigmoidal at 100 µM (Figure 1A, bottom left) and 1 mM mefenamic acid (data not shown [15]) suggesting that the residue K41 in KCNE1 is critical to the potentiation action of mefenamic acid.

Since the presence of four mutant K41C-KCNE1 subunits was shown to abolish the effect of mefenamic acid and a total of between one and four KCNE1 subunits are known to co-assemble with the KCNQ1 channel complex, we then examined whether the action of mefenamic acid was impacted by introducing different ratios of mutant K41C- and WT KCNE1 subunits into a fully saturated *I*_*Ks*_ channel complex. K41C and WT KCNE1 subunits were introduced in a ratio of 3:1 (EQQQQ, transfected with K41C-KCNE1), 2:2 (EQQ, transfected with K41C-KCNE1), and finally 1:3 (K41C-EQQQQ, transfected with WT KCNE1) K41C-KCNE1 to WT KCNE1 subunits (see Materials and Methods). In all three cases, fully saturated *I*_*Ks*_ channel complexes will comprise a 4:4 ratio of KCNE1:KCNQ1.

When three out of the four subunits were mutated (EQQQQ + K41C-KCNE1), no significant changes to the shape or V_1/2_ of the G-V relationship were seen after treatment with 100 µM mefenamic acid (Figure 1B, right, and Table 1). Additionally, the EQQQQ + K41C-KCNE1 current waveform remained largely unaffected by the drug at this concentration. A sigmoidal current waveform both in the absence (Figure 1B, top left) and presence of 100 µM mefenamic acid (Figure 1B, bottom left) was observed. The tail current decay also remained rapid, unlike in WT EQ (Figure 1A, top left) where complete inhibition of tail current decay was observed after treatment with 100 µM mefenamic acid. Thus, EQQQQ + K41C-KCNE1 constructs failed to display any of the three hallmarks of mefenamic acid (changes to the current waveform, hyperpolarization of the V_1/2_ and shape change of the G-V plot) at a dose of 100 µM. However, at concentrations of mefenamic acid above 100 µM, the sigmoidal EQQQQ + K41C-KCNE1 waveform was transformed into one with drastically faster onset and partially inhibited tail current decay (Figure 2A). Mefenamic acid reached its maximum efficacy at 500 µM (Figure 2A). At the highest concentration of 1 mM mefenamic acid, the normalized response was approximately 65% that of WT EQ which indicates decreased efficacy and a heavily right-shifted dose–response relationship, but one that was still responsive to mefenamic acid (EQQQQ + K41C-KCNE1 EC_50_ = 262 μM; WT EQ EC_50_ = 12.4 μM; Figure 2C).

The introduction of 2:2 K41C-KCNE1 to WT KCNE1 subunits (EQQ + K41C-KCNE1) produced the same results as EQQQQ + K41C-KCNE1 when treated with 100 µM mefenamic acid. The slope and V_1/2_ of the EQQ + K41C-KCNE1 G-V relationship remained almost the same after treatment with 100 µM mefenamic acid (Figure 1C, right, and Table 1). A sigmoidal waveform with tail current decay was also seen both in the absence (Figure 1C, top left) and presence of the drug (Figure 1C, bottom left). EQQ + K41C-KCNE1 at increasingly higher concentrations of mefenamic acid also responded to the drug in a dose-dependent manner (Figure 2B), and the response of EQQ + K41C-KCNE1 was greater than that of EQQQQ + K41C-KCNE1 (EQQQQ + K41C-KCNE1 EC_50_ = 262 μM; EQQ + K41C-KCNE1 EC_50_ = 173 μM, Figure 2B,C). At the highest concentration of mefenamic acid tested, 1 mM, instantaneous current onset and complete inhibition of tail current decay was seen with EQQ + K41C-KCNE1 (Figure 2B). This mefenamic acid-enhanced EQQ + K41C-KCNE1 current was subsequently fully inhibited by 1 µM of the specific *I*_*Ks*_ inhibitor, HMR1556 (Figure 2B, green trace). These results suggest that at least two WT KCNE1 subunits within the I_Ks_ complex are necessary to maintain maximal efficacy of mefenamic acid.

Finally, the introduction of only one mutant subunit in a fully saturated channel complex (K41C-EQQQQ + KCNE1) only partially prevented the three hallmarks of mefenamic acid at a dose of 100 µM. Although the V_1/2_ was hyperpolarized (ΔV_1/2_ = −59.9 mV) and the slope of the G-V curve was decreased for K41C-EQQQQ + KCNE1 in the presence of 100 µM mefenamic acid compared to control (control *k*, slope factor = 27.6 mV; Mef *k*, slope factor = 44.5 mV; Figure 1D, right and Table 1), both of these effects were less than that of WT EQ (Figure 1A right; circles). Despite reducing changes in the G-V relationship, the presence of only one mutant K41C-KCNE1 subunit did not prevent changes to the current waveform. An instantaneous current onset with complete inhibition of tail current decay was seen after treatment of K41C-EQQQQ + KCNE1 with 100 µM mefenamic acid (Figure 1D, bottom left). Furthermore, at all concentrations tested, the response of K41C-EQQQQ + KCNE1 to mefenamic acid was comparable to that of WT EQ (Figure 2C).

Overall, the results above indicate that the ability of K41C to prevent the activating effect of mefenamic acid is stoichiometrically graded in a non-linear manner. The fewer mutant K41C β-subunits present in the fully saturated *I*_*Ks*_ complex, the more effective mefenamic acid was in activating *I*_*Ks*._ Additionally, the dramatic shift in V_1/2_ seen when WT EQ was treated with 100 µM mefenamic acid was found to be absent or reduced in a graded manner, when channel complexes contained four, three, and two K41C-KCNE1 subunits (Figure 2D). K41C-EQQQQ + KCNE1 still showed a significant shift in V_1/2_ suggesting that one mutant K41C-KCNE1 subunit in a saturated *I*_*Ks*_ channel complex is not enough to prevent the channel from being fully activated by 100 µM mefenamic acid. Importantly, at higher concentrations than 100 µM, all constructs except EQQQQ + K41C-KCNE1, which contained only one WT KCNE1 subunit, could be activated to the level of WT in the presence of 1 mM mefenamic acid.

### Varying the number of K41C-containing subunits in models of mefenamic acid activation of I_Ks_

**Figure 3.**
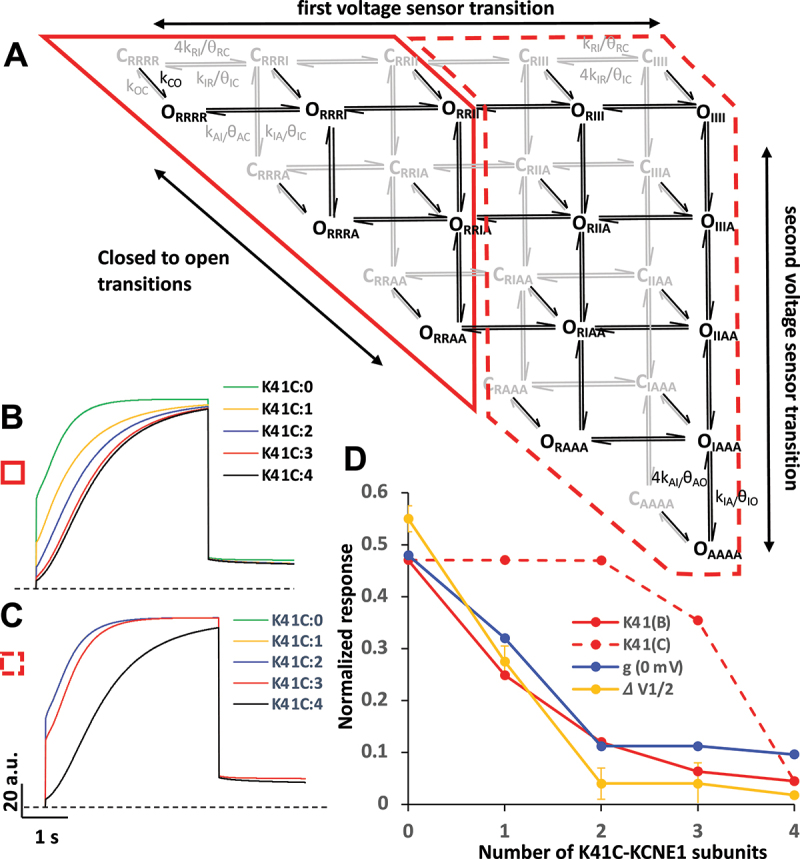
Model of mefenamic acid action in K41C mutants.

In order to explore how inclusion of different numbers of K41C-KCNE1 mutant subunits modifies drug action, a model of *I*_*Ks*_ channel activity that we have used previously was modified to allow the kinetics of individual VS to simulate the WT channel response or K41C channel insensitivity to mefenamic acid (Figure 3). The model scheme is shown in Figure 3A, and further details, including the rates for all transitions may be found in the Materials and Methods in its original published derivation [16]. Essentially, the model tracks the movement of four independent VS from closed resting states (C_RRRR_) to fully activated open states (O_AAAA_). A quantitative simulation of the WT response to mefenamic acid is obtained by speeding the forward rates of each of the two VS transitions (see Materials and Methods [13]), as shown in the current simulations with zero K41C-KCNE1 mutant subunits (Figure 3B, K41C:0, i.e. WT) where 100 µM mefenamic acid induces an instantaneous current of about 50% of the peak activating current. WT and mutant K41C subunits were included in the model to simulate two extreme possibilities, as shown by the red-delineated areas in the model scheme, where WT VS transitions either dominate the early activation pathway of the channels (red solid outline area), and K41C mutant subunits underlie the later VS transitions (red dash outline area), or vice-versa. The results of the simulations under these two conditions are quite different. We expect WT subunits to activate first in the presence of mefenamic acid, and the effect of a single K41C subunit is to reduce the response to 100 µM mefenamic acid by 50%, and then to 10% or less with the addition of further mutant VS (Figure 3B). As a test of the sensitivity of the model, placement of the WT subunits further to the right in the initial row of the activation pathway, blunts the effects of K41C mutants such that the activating response to mefenamic acid only starts to decline in the presence of three or more K41C-KCNE1 subunits (Figure 3C).

(A) Model of *I*_*Ks*_. Closed-state transitions are gray, open-state are black. C-O transitions and their reverse as black/gray arrows. VS state subscripts: *R* = rest; I = intermediate-activated; and A is the activated state. Only open states with activated VS conduct current. Selected transition rates are shown, for other rates, see Methods. Red boxes divide VS movement into two early steps (solid) and two later steps (dashed). (B) Simulation of *I*_Ks_ currents at +80 mV in 100 mM Mef. Inset denotes number of K41C-KCNE1 subunits in a 4:4 complex, so K41C:0 = WT. WT subunits were added to model starting at the left of the scheme, replacing K41C subunits one at a time (red outline area in A). Dotted line represents zero current. (C) As for B, except that WT subunits were added from the right side of the scheme (dashed red outline). (D) Model Mef response (red curves) *vs*. number of K41C-KCNE1 subunits compared with conductance (at 0 mV, Figure 1) and ∆V_1/2_ (Figure 2D) responses to Mef. The data are normalized to WT response of 100 mM Mef.

As WT VSs have augmented kinetics in the presence of mefenamic acid, it is expected that they will activate before K41C subunits, so the first scenario described above with WT subunits activating first (area circumscribed by solid red line) is the most likely and fits the experimental data much better (Figure 3D). This model response to mefenamic acid simulates well the changes in conductance at 0 mV (Figure 1) and the ΔV_1/2_ (Figure 2D) induced by 100 µM mefenamic acid in channel complexes that contain increasing numbers of K41C-KCNE1 subunits.

## Discussion

### Insights from K41C-KCNE1 on mefenamic acid action

In a previous study, we showed that mefenamic acid has little to no effect on KCNQ1 alone, which suggested that the drug required the presence of KCNE1 to either bind and/or facilitate its effect on the channel, and also that the effect was dependent on the stoichiometric ratio of KCNE1:KCNQ1 subunits [13]. The more saturated the *I*_*Ks*_ channel complex was, the greater the effect of mefenamic acid (1 KCNE1 subunit in the channel complex < 2 KCNE1 subunits < 4 KCNE1 subunits) intimating that the binding of each KCNE1 subunit to the channel in turn forms a binding site for the drug. A total of four identical mefenamic acid binding or regulatory pockets should be available on the *I*_*Ks*_ channel complex. Consistent with this idea, when all four WT KCNE1 subunits are replaced with mutant K41C-KCNE1 subunits, mefenamic acid up to a concentration of 1 mM is ineffective (Figure 1A). No current waveform changes, slope changes to the G-V plot, or V_1/2_ shifts were seen, suggesting that all the drug binding site(s) on the channel complex were impaired, or that the mechanism of action was disabled. Further detailed studies of mefenamic acid binding to *I*_*Ks*_ channel complexes are the subject of a recent study from our laboratory, the results of which support the conclusions above [15].

A closer examination of results from channel complexes with different mutant to WT KCNE1 ratios reveals that K41C-EQ (4:0, Figure 1A; triangles), EQQQQ + K41C-KCNE1 (3:1, Figure 1B,) and EQQ + K41C-KCNE1 (2:2, Figure 1C) all prevented the effects of 100 μM mefenamic acid. Only treatment of K41C-EQQQQ + KCNE1 (1:3) with 100 μM mefenamic acid allowed instantaneous current onset, shape change to the G-V and a V_1/2_ shift (Figure 1D, Table 1). This G-V plot shape change and V_1/2_ shift were still less than that seen after treatment of WT EQ with the same concentration of mefenamic acid, suggesting that four mefenamic acid molecules likely bind to and act on the WT EQ channel complex at the same time. The binding of four drug molecules is consistent with the presence of a total of four identical mefenamic acid binding pockets formed between KCNQ1 and KCNE1 [15].

When one to four K41C-KCNE1 subunits are replaced with WT KCNE1 subunits, the effect of 100 μM mefenamic acid is brought back in a stoichiometrically graded manner. Consistent with EQQ + K41C-KCNE1 (2:2 ratio of mutant to WT KCNE1 subunits) having one more WT binding site than EQQQQ + K41C-KCNE1 (3:1) and K41C-EQQQQ + KCNE1(1:3) having yet another additional WT binding site, the EC_50_ for EQQQQ + K41C-KCNE1 was found to be the greatest followed by that of EQQ + K41C-KCNE1 then finally K41C-EQQQQ + KCNE1 (EQQQQ + K41C-KCNE1 EC_50_ = 262 μM; EQQ + K41C-KCNE1 EC_50_ = 173 μM; K41C-EQQQQ + KCNE1 EC_50_ = 13.3 μM) (Figure 2C). In the case of K41C-EQQQQ + KCNE1 (1:3) the EC_50_ remains relatively unchanged (EC_50_ = 13.3 μM) from WT EQ (EC_50_ = 12.8 μM, Figure 2C), although one mutant K41C-KCNE1 is present in the channel complex, the three remaining WT binding sites are enough to overcome the effect of the mutation. The overlap of both WT EQ and K41C-EQQQQ + KCNE1 dose response curves is consistent with the expected incorporation of four KCNE1 subunits in the complex. The EC_50_ of EQQ + K41C-KCNE1 (2:2, EC_50_ = 173 μM) and EQQQQ + K41C-KCNE1 (3:1, EC_50_ = 262 μM) dose–response curves are significantly different from WT EQ (95% confidence intervals do not overlap, see Figure 2C legend). The K41C mutation may alter the overall conformation of the remaining binding pockets in the adjacent subunits, which results in a different EC_50_ compared to the WT binding pocket [19]. The EQQ + K41C-KCNE1 and EQQQQ + K41C-KCNE1 concentration–response curves may also give insight into the mechanism of action of mefenamic acid. At lower concentrations, below 100 μM, mefenamic acid has little effect on these complexes. Since we now understand that the K41C mutation destabilizes drug binding [15], it is possible that below 100 μM, the unbinding rate may exceed the drug on rate, which is a function of concentration. However, once the mefenamic acid concentration is high enough, the destabilization induced by K41C is overcome, and the response to the drug then increases as a function of concentration. This mass action effect of the drug may also explain why, when sufficient drug is present, the efficacy of mefenamic acid is the same as WT in EQQ + K41C-KCNE1. Only the limiting solubility of the drug prevented us from testing this hypothesis in EQQQQ + K41C-KCNE1, and indeed in K41C-EQ as well.

It is worth noting that we have recently shown that high concentrations of mefenamic acid can inhibit the currents from WT-EQ and some mutated EQ channels [18]. However, this inhibition has not been realized nor quantified in constructs carrying the K41C mutation, particularly in the EQQ + K41C-KCNE1 construct (Figure 2B). Previous studies showing inhibitory effects of mefenamic acid have typically involved saturated *I*_Ks_ molecular complexes (4:4 stoichiometry of either WT or mutated channels) compared to the mixed configuration used here. It is conceivable that the inhibitory binding site, or the conformational state that favors inhibition, is either absent or sterically hindered in the presence of K41C-KCNE1 subunits combined with the WT KCNE1 + KCNQ1 concatemers. Another possibility is that the K41C mutation itself alters the local environment in a way that reduces the ability of mefenamic acid to access or stabilize an inhibitory conformation. The absence of inhibition at 100 μM, as shown in Figure 2B, may reflect a shift in the balance between activating and inhibitory effects, with the activation-dominant response out-competing a potentially weak or absent inhibitory component. It should be noted that, even if present, current inhibition would not preclude normalized responses from reaching the maximum potentiation effect of 1.0. Rather, the dose–response curve would approach the maximum response at lower concentrations due to the reduction in end of pulse current amplitude.

Simulation of the action of mefenamic acid on individual K41C subunits using the same model configurations that reproduced its characteristic drug action on WT *I*_*Ks*_ channels [13] reproduced the new experimental data well. The model suggested that WT subunits, with accelerated VS activation kinetics in the presence of the drug outcompeted K41C-containing subunits early in the activation pathway, so that later activating K41C subunits which determined the activation kinetics of the final channel open states were able to delay opening when one or more of them were present in the channel complex.

The importance of residue K41 to the binding of mefenamic acid is further supported by the dependence of the mefenamic acid effect on the number of available binding sites. Two to four K41C containing KCNE1 subunits largely prevented the actions of mefenamic acid, reducing the maximum response by 50–100%. One K41C-KCNE1 subunit was found to be insufficient to fully prevent the effect of 100 µM mefenamic acid, which when compared to WT EQ in the presence of the drug suggested that four drug molecules can likely bind to the WT *I*_*Ks*_ channel complex at once.

## Data Availability

Published data are available upon request from the corresponding author.

## References

[1] Nakajo K, Ulbrich MH, Kubo Y, et al. Stoichiometry of the KCNQ1-KCNE1 ion channel complex. Proc Natl Acad Sci USA. 2010;107(44):18862–10. doi: 10.1073/pnas.1010354107 20962273 PMC2973890

[2] Dvir M, Strulovich R, Sachyani D, et al. Long QT mutations at the interface between KCNQ1 helix C and KCNE1 disrupt I(KS) regulation by PKA and PIP₂. J Cell Sci. 2014;127(Pt 18):3943–3955. doi: 10.1242/jcs.147033 25037568 PMC6519428

[3] Murray CI, Westhoff M, Eldstrom J, et al. Unnatural amino acid photo-crosslinking of the IKs channel complex demonstrates a KCNE1: KCNQ1 stoichiometry of up to 4:4. Elife. 2016;5. doi: 10.7554/eLife.11815 PMC4807126 26802629

[4] Wrobel E, Tapken D, Seebohm G. The KCNE tango - how KCNE1 interacts with Kv7.1. Front Pharmacol. 2012;3:142. doi: 10.3389/fphar.2012.00142 22876232 PMC3410610

[5] Wang K, Terrenoire C, Sampson KJ, et al. Biophysical properties of slow potassium channels in human embryonic stem cell derived cardiomyocytes implicate subunit stoichiometry. J Physiol. 2011;589(Pt 24):6093–6104. doi: 10.1113/jphysiol.2011.220863 22025662 PMC3286688

[6] Yu H, Lin Z, Mattmann ME, et al. Dynamic subunit stoichiometry confers a progressive continuum of pharmacological sensitivity by KCNQ potassium channels. Proc Natl Acad Sci USA. 2013;110(21):8732–8737. doi: 10.1073/pnas.1300684110 23650380 PMC3666726

[7] Gao Z, Xiong Q, Sun H, et al. Desensitization of chemical activation by auxiliary subunits: convergence of molecular determinants critical for augmenting KCNQ1 potassium channels. J Biol Chem. 2008;283(33):22649–22658. doi: 10.1074/jbc.M802426200 18490447 PMC2504881

[8] Magyar J, Horvath B, Banyasz T, et al. L-364,373 fails to activate the slow delayed rectifier K+ current in canine ventricular cardiomyocytes. Naunyn Schmiedebergs Arch Pharmacol. 2006;373(1):85–9. doi: 10.1007/s00210-006-0047-4 16544107

[9] Toyoda F, Ueyama H, Ding WG, et al. Modulation of functional properties of KCNQ1 channel by association of KCNE1 and KCNE2. Biochem Biophys Res Commun. 2006;344(3):814–820. doi: 10.1016/j.bbrc.2006.03.213 16631607

[10] Busch AE, Herzer T, Wagner CA, et al. Positive regulation by chloride channel blockers of I sK channels expressed in Xenopus oocytes. Mol Pharmacol. 1994;46(4):750–753. doi: 10.1016/S0026-895X(25)09808-6 7969055

[11] Abitbol I, Peretz A, Lerche C, et al. Stilbenes and fenamates rescue the loss of I KS channel function induced by an LQT5 mutation and other IsK mutants. Embo J. 1999;18(15):4137–4148. doi: 10.1093/emboj/18.15.4137 10428953 PMC1171491

[12] Unsöld B, Kerst G, Brousos H, et al. KCNE1 reverses the response of the human K + channel KCNQ1 to cytosolic pH changes and alters its pharmacology and sensitivity to temperature. Pflugers Arch Eur J Physiol. 2000;441(2–3):368–378. doi: 10.1007/s004240000434 11211125

[13] Wang Y, Eldstrom J, Fedida D. The I Ks ion channel activator mefenamic acid requires KCNE1 and modulates channel gating in a subunit-dependent manner. Mol Pharmacol. 2020;97(2):132–144. doi: 10.1124/mol.119.117952 31722973

[14] Xu X, Jiang M, Hsu KL, et al. KCNQ1 and KCNE1 in the IKs channel complex make state-dependent contacts in their extracellular domains. J Gen Physiol. 2008;131(6):589–603. doi: 10.1085/jgp.200809976 18504315 PMC2391252

[15] Chan M, Sahakyan H, Eldstrom J, et al. A generic binding pocket for small molecule I(Ks) activators at the extracellular inter-subunit interface of KCNQ1 and KCNE1 channel complexes. Elife. 2023;12:1–31. doi: 10.7554/eLife.87038 PMC10501768 37707495

[16] Westhoff M, Eldstrom J, Murray CI, et al. I Ks ion-channel pore conductance can result from individual voltage sensor movements. Proc Natl Acad Sci USA. 2019;116(16):7879–7888. doi: 10.1073/pnas.1811623116 30918124 PMC6475427

[17] Santiago-Castillo JA, Covarrubias M, Sanchez-Rodriguez JE, et al. Simulating complex ion channel kinetics with IonChannelLab. Channels (Austin). 2010;4(5):422–428. doi: 10.4161/chan.4.5.13404 20935453 PMC3051876

[18] Chan M, Pourrier M, Eldstrom J, et al. Dual effects of mefenamic acid on the I(Ks) molecular complex. Br J Pharmacol. 2025;182(4):1075–1089. doi: 10.1111/bph.17389 39520043

[19] Cattoni DI, Chara O, Kaufman SB, et al. Cooperativity in binding processes: new insights from phenomenological modeling. PLOS ONE. 2015;10(12):e0146043. doi: 10.1371/journal.pone.0146043 26717487 PMC4696654

